# Trends in microbial profile of burn patients following an event of dust explosion at a tertiary medical center

**DOI:** 10.1186/s12879-020-4920-4

**Published:** 2020-03-04

**Authors:** Yin-Yin Chen, Ping-Feng Wu, Chii-Shya Chen, Ian-Horng Chen, Wan-Tsuei Huang, Fu-Der Wang

**Affiliations:** 1 Department of Infection Control, Taipei Veterans General Hospital, Taipei, Taiwan; 2 Department of Nursing, Taipei Veterans General Hospital, Taipei, Taiwan; 3 College of Nursing, National Yang–Ming University, Taipei, Taiwan; 4 Division of Infectious Diseases, Department of Medicine, Taipei Veterans General Hospital, Taipei, Taiwan; 5 School of Medicine, National Yang-Ming University, Taipei, Taiwan; 6 Taipei City Hospital, Taipei, Taiwan; 7 Institute of Public Health, and Community Medicine Research Center, National Yang-Ming University, Taipei, Taiwan

**Keywords:** Burn, Infection, Microbial

## Abstract

**Background:**

Microbial infection is the main cause of increased morbidity and mortality in burn patients, especially infections caused by multiple drug-resistant organisms (MDRO). The purpose of this study was to explore major microbial trends in burn patients**.**

**Methods:**

This retrospective study was conducted at burn wards and intensive care units, where burn patients were admitted following an event of dust explosion. Data were collected for a number of variables including severity of burns, demographic and clinical characteristics, laboratory data, and therapeutic devices.

**Results:**

A total of 1132 specimens were collected from 37 hospitalized burn patients with mean TBSA of 46.1%.The most commonly isolated species were *Staphylococcus spp.* (22.4%). The highest rate of antibiotic resistance was observed in carbapenem–resistant *A. baumannii* (14.6%), followed by methicillin-resistant *S. aureus* (11.3%). For each additional 10% TBSA, the isolation of MDRO increased 2.58–17.57 times (*p* < 0.05); for each additional 10% of the third-degree burn severity, the risk of MDRO significantly decreased by 47% (95% CI, 0.38–0.73, *p* < 0.001) by Cox model.

**Conclusions:**

The proportion of overall microbial isolates increased with the increase in TBSA and duration of time after burns. The extent of TBSA was the most important factor affecting MDRO.

## Background

Generally, microorganisms will colonize and grow quickly after burns due to the loss of the skin barrier [1]. In burn patients, potential biomarkers can be used clinically to identify infections and sepsis; they can also be used to predict the survival of injuries, monitor the severity of injuries, organ function or wound healing. There are several risk factors which facilitate microbial colonization and infection, including age and comorbidities, burn wound size, impaired immunity (e.g., hyperglycemia, hypermetabolic response), and medical measures (e.g., use of invasive catheters, transfusion, delays in burn wound excision) etc. [1, 2]. The microbial colonizers or pathogens affecting burn patients include bacteria and fungi [3, 4]. The most common Gram-positive bacteria implicated in burn wound infections include *Staphylococcus spp.*, *Enterococcus spp.*, and beta-hemolytic *Streptococcus* group A. Among that group, *Staphylococcus aureus* continues to be one of the most important bacterial cause of burn wound infections [4–7]. The most frequently isolated Gram-negative bacteria from patients with burn wounds include *Pseudomonas aeruginosa*, *Acinetobacter baumannii*, *Klebsiella* spp*.*, *Stenotrophomonas* spp*.*, *Escherichia coli*, and *Enterobacter cloacae* [1, 2, 5, 7, 8].

Patients with severe burns are more prone to infections caused by multiple drug-resistant organisms (MDRO); common examples include methicillin-resistant *Staphylococcus aureus* (MRSA), vancomycin-resistant *Enterococcus* (VRE), MDR *Pseudomonas* spp*.* and *Acinetobacter* spp*.* [5, 9]. The care and treatment of these patients have been quite challenging. If some of the infection control measures are neglected, these pathogens may even cause an outbreak in burn units [3, 10]. These infections result in prolonged hospital stay and high mortality rates in patients with burns. Additionally, the cost of medical care for burn patients is substantial [11–13].

A number of previous studies used databases to conduct and publish clinical epidemiological data on populations of burn patients that mostly involved groups of children with varying degrees of burns [14–17]. Thus, the population analysis of these different databases exhibited more heterogeneity. One sudden dust (flammable starch-based powder) explosive event occurred at a recreational water park in northern Taiwan. More than 4000 young attendees went to the music party where the incident happened on stage. As a result, 499 people were injured, 393 were hospitalized and 221 of them ended up in the intensive care units (ICUs) of 46 hospitals in seven cities. As most attendees wore flammable swimwear, the patients had large total body surface area (TBSA, average 41%) of burns. A total of 281 people suffered more than 40% injuries; of those, 41 victims were severely injured with TBSA more than 80%, and 15 fatalities were attributed to the explosion [18]. Among the hospitalized, 44 patients with similar age group and exposure levels were enrolled in the study.

There have been limited published reports addressing changes in trends among various important microorganisms isolated from burn patients. We hypothesized that various important microorganisms and their MDRO would show different trends due to differences in burn severity and time. Therefore, the purpose of our study was to determine the isolation rates of common microorganisms and MDRO, and their fluctuations and trends in TBSA range as well as post-burn times using stratified trends and multiple analyses.

## Methods

### Hospital and cases

This retrospective observational study, using an active hospital–wide HAI surveillance from June 27, 2015 to October 31, 2015 was conducted at a burn department (5 bed–ICU and 14 bed–ward), four other ICUs of a 3045–bed tertiary referral medical center in northern Taiwan. The study protocol was approved by the Institutional Review Board of the research hospital, and the requirement for obtaining informed consent was waived.

After the event of the dust explosion on the evening of June 27, 2015, 44 injured patients were immediately sent to our emergency department for medical treatment. Eight victims were discharged after treatment for minor wounds, while 36 victims with severe burns needed to be hospitalized for treatment and care. On the following day, hospitalized burn cases were further evaluated for graded assessment of the severity of burns. Afterwards, the case beds were rearranged from the original emergency hospitalization to the dispersal of wards (16 ICUs or wards) and to centralized ward care (5 ICUs and 1 ward). Two cases were transferred from other hospitals on June 28 and 29 and one victim was transferred to another hospital on July 1. All patients hospitalized from a period of June 27 to 29, 2015 due to the dust explosion were eligible for the study. Those cases that were discharged from the hospital, but readmitted later, were excluded from the study.

### Strategies for infection control

When patients were transferred to the emergency department, an interdisciplinary medical team was quickly established and held regular meetings on treatment and disposal strategies. An infectious disease physician also participated in the antimicrobial stewardship including assessments and medications. In addition, sufficient healthcare workers and appropriate administrative support and management staff was provided.

Cases underwent escharotomy or debridement to remove damaged tissue in operation room. Human skin or fish skin were provided for graft to treat severe burns. The choice of dressing type was determined by the attending physician of the interdisciplinary team depending on the depth, size, exudate, and infection of the burn wound. For example, AQUACEL dressings (ConvaTec Inc., UK) were used for large-scale third burn-degree; and AQUACEL Ag + dressings which the power to disrupt and destroy biofilm were used for infected wounds. Dressing changes for all cases, if required, were carried out at their bedsides.

The prevention and control practices for burn infections have been devised according to recommendations from published guidelines [1, 2, 9]. The healthcare personnel and visitors were required to strictly adhere to the infection control procedures. Protocols were also developed in accordance with standard procedures for environmental cleaning, disinfection procedures, and training of housekeeping staff to perform cleaning and disinfection. During this study, the infection control practitioners (ICPs) used a checklist to improve the auditing of the cleaning personnel’s correct implementation of equipment and the environment cleaning and disinfection. An adenosine triphosphate (ATP) luminometer was also used to measure the level of cleanliness. When the ATP measurements were lower than the cleaning standard value (< 100 relative light unit), the microbial surveillance of the indoor environment and the equipment would be carried out, and it must be cleaned again until the cleaning standard value were met. Additionally, a laboratory-based alert system was established that immediately notified ICPs and clinical personnel when new MDRO were isolated from cases.

### Data collection

Data on the severity of burns and frequencies of surgery, demographic and clinical characteristics, laboratory data and therapeutic devices used were recorded. Additionally, revised Baux score, calculated as the sum of the age and TBSA was also included. Baux scores were increased by 17 and 0 for all cases with inhalation injuries and without inhalation injuries, respectively (see Tables 1 and 2). If no clinically significant microorganism was isolated, empirical antimicrobial treatment, such as a carbapenem plus glycopeptide, was administered for < 7 days to hospitalized patients with severe sepsis or septic shock and a large burn area [19]. Antifungal agent (fluconazole or echinocandin, if fluconazole was contraindicated) was administered at 7 days after the burn, if the patient exhibited at least three specific risk factors.

**Table 1:** Distribution of severity of burns and frequencies of surgery

Variables	Number (***n*** = 37)	Percentage (%)
Number of total body surface area (%) (mean ± SD; range)	46.1 ± 29.1	5–92
< 10	6	16.2
10–19	4	10.8
20–29	2	5.4
30–39	1	2.7
40–49	5	13.5
50–59	6	16.2
60–69	3	8.1
70–79	4	10.8
80–89	2	5.4
> 90	4	10.8
Number of third -degree burn severity (%)		
< 10	3	8.1
10–19	1	2.7
20–29	3	8.1
30–39	1	2.7
40–49	3	8.1
50–59	4	10.8
60–69	4	10.8
70–79	3	8.1
80–89	1	2.7
> 90	4	10.8
Main site of the third -degree burn		
30–50% burn of upper limb, except wrist and hand	18	48.6
30–50% burn of lower limbs	18	48.6
30–50% burn of trunk	15	40.5
30–50% burn of face, head, and neck	10	27.0
20–50% burn of wrists and hands	4	10.8
30–50% burn of multiple specified sites	4	10.8
Unspecified degree	10	27.0
Number of surgeries		
0	5	13.5
1	7	18.9
2	5	13.5
3	7	18.9
4	4	10.8
5	3	8.1
6	1	2.7
Number of various surgical procedures		
1	4	10.8
2	1	2.7
4	2	5.4
5	2	5.4
6	3	8.1
7	4	10.8
8	2	5.4
9	1	2.7
> 10	8	21.6
Main types of surgery (*n* = 142)		
Operations on skin and subcutaneous tissue	94	66.2
Operations on muscle, tendon, and fascia of hand	18	12.7
Other operations on vessels	11	7.7
Time spent of first surgery (mean ± SD; range) (minutes)	58.5 ± 40.7	5–215
Time spent of surgery during the 7 days before healthcare-associated infection (mean ± SD; range) (minutes)	76.3 ± 57.3	8–269

**Table 2:** Demographic and clinical characteristics of burn cases

Variables	Number (***n*** = 37)	Percentage (%)
Intensive care units	24	64.9
Gender		
Male	22	59.5
Female	15	40.5
Inhalation injury	32	86.5
**Laboratory data**^a^		
White blood cell (≥10,500 mm^3^)	27	73.0
Hematocrit < 35%	8	21.6
Serum sodium (≥146 mmol/L)	6	16.2
Serum potassium (≥5.5 mmol/L)	4	10.8
Serum creatinine (≥1.5 mg/dL)	4	10.8
Serum albumin (≤2.49 g/dL)	0	0.0
**Invasive devices (yes)**		
Endotracheal tube	23	62.2
Mechanical ventilator > 96 h	16	43.2
Central line catheter	18	48.6
Foley catheter	13	35.1
Hemodialysis	2	5.4
Extracorporeal membrane oxygenation	1	2.7
**Site of healthcare-associated infection (*n*** **= 40)**		
Bloodstream infection	28	70.0
Symptomatic urinary tract infection	9	22.5
Asymptomatic bacteriuria	2	5.0
Central nervous system infection	1	2.5
Mortality	1	2.7
**Continuity variables**	mean ± SD	min-max
Age (years)	22 ± 6.2	13–38
APACHE II scores	14.3 ± 8.0	2–32
LOS before the first isolated MDR (*n* = 23)	17.7 ± 16.0	2–55^a^
Total LOS in hospital stay^#^	85.4 ± 70.4	9–276^a^
Baux score	24.5 ± 8.7	6–46

*SD* standard deviation, *APACHE* acute physiology and chronic health evaluation, *LOS* length of stay, *MDR* multi-drug resistant microbes

^a^The highly abnormal values during the hospital stay were selected for the analysis

^#^The LOS is calculated throughout the hospital stay, and other data are within 3 months of hospitalization (from June 27 to October 31)

The standard surveillance protocols and healthcare-associated infection (HAI) site definitions were according to the National Healthcare Safety Network of the Centers for Disease Control and Prevention [20]. Culturing of pathogens was performed when the patient had a temperature over 38 °C or a fever of unknown etiology, exudates or pus from an insertion site or surgical site, or signs and symptoms of suspected infections. The microbial culture of burn wounds was judged by the physician for the degree of the wound dirty and the signs or symptoms of infection. Specimens of a burn wound were usually collected after the wound has been cleaned during the patient’s operation. Microbiological work was carried out in a microbiology laboratory that was certified internationally through the College of American Pathologists. Identification of all microbial isolates was confirmed at the laboratory using automated-method employing identification cards of VITEK-MS system (BioMerieux Inc., Mercy L’etoil, France). The susceptibility results were interpreted based on the criteria specified by the Clinical and Laboratory Standards Institute 2015. Cultures reported with intermediate sensitivity were considered resistant.

### Statistical analysis

A descriptive analysis of all collected variables was performed. For any given case if same species were isolated from the same site on multiple occasions, they were recorded once only. Microbiological profile was established including type of bacteria and antibiotic susceptibility. Stratified analyses of TBSA and days after admission were used. The Chi square test for linear trend was used to examine the trends in main microbial isolates in TBSA and days after admission. The simple logistic regression and the simple Cox regression analyses were also used. Next, the multiple logistic regression and the multiple Cox regression with an enter approach were used to assess risk factors of MDRO, while adjusting for potentially confounding variables such as TBSA, and burn-degree severity. Odds ratios (OR), hazard ratio (HR), and 95% confidence intervals (CI) were calculated. All tests were two-tailed, and *p* values less than 0.05 were considered statistically significant.

## Results

### Patient demographic data

A total of 37 severely hospitalized burn cases were admitted during the study period. Of these, 24 cases (64.9%) needed to stay in the ICUs for treatment. The mean percentage of initial TBSA was 46.1% ± 29.1% (median 50%, range 5–92%; > 50% 21 cases) and 16 cases had more than 50% third-degree burns (43.2%). The main sites with 30–50% of third-degree burns were upper limbs (except wrist and hand; 48.6%), lower limbs (48.6%), and trunk (40.5%). There were 4 cases with up to 90% of the TBSA and the third-degree burns. In total, 32 cases (86.5%) underwent surgery. Eight (21.6%) cases received more than 10 surgical procedures. The most common types of surgeries were operations on skin and subcutaneous tissues (66.2%). The mean time spent of first surgery had 58.5 ± 40.7 min and surgery during the 7 days before HAI had 76.3 ± 57.3 min (Table 1).

The mean age of the hospitalized burn cases was 22.0 ± 6.2 (range 13–38) years including 22 (59.5%) males. Mean APACHE score was 14.3 ± 8 points; 10.8% had abnormal serum creatinine levels (≥ 1.5 mg/dL). There were 23 (62.2%) cases with endotracheal tubes. Mean LOS before the first isolated MDRO (*n* = 23) was 17.7 ± 16 days. In total, 17 (45.9%) cases developed HAI with 40 episodes. The most common type of HAI was bloodstream infections (BSIs; 70%) with 28 episodes (Table 2). The mean Baux score was 24.5 ± 8.7 points with mortality rate of 2.7%. One case (70% TBSA and 49% third-degree burns) died with Baux score of 31 points, 4 months after admission.

### Microbiological investigations

Table 3 lists the distribution of microbial isolates by TBSA and by days after admission. Out of 1132 clinical specimens, 706 were positive in cultures (62.4%). A total of 335 strains of bacteria were isolated in the study. The three most commonly isolated groups of microorganisms were Gram-negative bacilli (GNB, *n* = 178, 53.1%) including glucose non-fermenting Gram-negative bacilli (GNFGNB, *n* = 132, 39.4%); Gram–positive bacteria (*n* = 101, 30.2%) including Gram–positive cocci (GPC, *n* = 97, 29%); and fungus (*n* = 51, 15.2%). The most commonly isolated species include *Staphylococcus* spp*.* (*n* = 75, 22.4%), *Acinetobacter* spp*.* (*n* = 63, 18.8%), and yeast (*n* = 45, 13.4%). In addition, only 1.2% (*n* = 4) of anaerobes were isolated. The MDRO accounted for 31% of all microbial isolates. The isolated MDROs began to increase in patients with TBSA ≥50% and hospitalization ≥15 days. The most commonly isolated MDRO include carbapenem–resistant *A. baumannii* (CRAB, 14.6% [imipenem–resistant 10.7% and pandrug-resistant 3.9%]); MRSA (11.3%); and carbapenem–resistant *Klebsiella pneumoniae* (CRKP, 2.4%). In addition, pandrug-resistant *P. aeruginosa* (PDRPA, 0.3%) was isolated from one case of more than 70% TBSA and hospitalized for more than 31 days.

**Table 3:** Distribution of microbial isolates by total body surface area and by days after admission

Isolated pathogens	Total	%	Total body surface area (%)							days after admission				
			< 39	40–49	50–59	60–69	70–79	80–89	> 90	1–3	4–7	8–14	15–30	> 31
**Total sampling numbers of culture specimen**	**706**		**28**	**65**	**118**	**108**	**151**	**142**	**94**	**66**	**38**	**78**	**162**	**362**
**Total number of culture-positive sample**	**335**	**100**	**6**	**29**	**43**	**58**	**84**	**62**	**54**	**26**	**28**	**38**	**92**	**151**
Rate of culture-positive (%)	47.5		21.4	44.6	36.4	53.7	55.6	43.7	57.4	39.4	73.7	48.7	56.8	41.7
**Total number of MDRO**	**104**		**1**	**9**	**17**	**21**	**20**	**19**	**17**	**2**	**6**	**9**	**35**	**52**
Rate of MDRO in total culture-positive (%)	31.0		0.96	8.7	16.3	20.2	19.2	18.3	16.3	1.9	5.8	8.7	33.7	50.0
**Gram–positive cocci**	**97**	**29.0**	**4**	**11**	**16**	**18**	**21**	**11**	**17**	**7**	**3**	**12**	**26**	**49**
**Staphylococcus sp.**	**75**	**22.4**	**3**	**10**	**12**	**15**	**13**	**8**	**14**	**2**	**3**	**11**	**23**	**36**
*S. aureus*	2	0.6	1	1	0	0	0	0	0	0	0	1	0	1
*S. aureus* (MRSA)	38	11.3	1	4	8	9	4	6	6	0	2	1	11	24
*coagulase–negative staphylococcus*	21	6.3	1	2	3	4	6	1	4	2	1	7	6	5
other *Staphylococcus sp.*	14	4.2	0	3	1	2	3	1	4	0	0	2	6	6
*Enterococcus* sp.	9	2.7	1	1	2	3	2	0	1	4	0	1	0	4
*E. faecium* (VRE)	7	2.1	0	0	1	0	4	2	0	1	0	0	2	4
Others	6	1.8	0	0	1	0	2	1	2	0	0	0	1	5
**Gram–positive bacilli**	**4**	**1.2**	**0**	**0**	**3**	**0**	**1**	**0**	**0**	**2**	**2**	**0**	**0**	**0**
**Gram–negative bacilli**	**46**	**13.7**	**1**	**3**	**7**	**7**	**11**	**8**	**9**	**4**	**3**	**7**	**12**	**20**
*Enterobacteriaceae*														
*Klebsiella pneumoniae*	9	2.7	0	0	3	2	1	2	1	0	1	1	3	4
*Klebsiella pneumonia* (CRKP)	8	2.4	0	0	0	4	1	1	2	0	0	1	2	5
*Escherichia coli*	5	1.5	0	1	0	0	1	1	2	0	0	0	3	2
*Enterobacter* sp.	4	1.2	1	1	0	0	2	0	0	1	1	0	1	1
*E. cloacae* (CRE)	1	0.3	0	0	1	0	0	0	0	1	0	0	0	0
*Proteus mirabilis*	4	1.2	0	0	1	0	2	0	1	0	0	0	2	2
*Serratia marcescens*	3	0.9	0	0	1	0	1	0	1	2	0	0	1	0
*Aeromonas hydrophila*	2	0.6	0	1	0	0	1	0	0	0	1	1	0	0
Others	10	3.0	0	0	1	1	2	4	2	0	0	4	0	6
**Non-fermentative Gram-negative bacilli**	**132**	**39.4**	**0**	**10**	**17**	**22**	**32**	**27**	**24**	**10**	**14**	**13**	**44**	**51**
***Acinetobacter sp.***	**63**	**18.8**	**0**	**8**	**9**	**12**	**13**	**12**	**9**	**3**	**6**	**9**	**26**	**19**
*A. baumannii*	1	0.3	0	0	0	1	0	0	0	0	1	0	0	0
*A. baumannii* (IRAB)	36	10.7	0	5	5	8	6	6	6	0	3	7	18	8
*A. baumannii* (PDRAB)	13	3.9	0	0	2	0	4	4	3	0	1	0	2	10
*A. baumannii* complex	13	3.9	0	3	2	3	3	2	0	3	1	2	6	1
*Pseudomonas aeruginosa*	33	9.9	0	0	3	5	10	6	9	2	3	0	10	18
*P. aeruginosa* (PDR-PA)	1	0.3	0	0	0	0	1	0	0	0	0	0	0	1
*Stenotrophomonas maltophilia*	13	3.9	0	1	2	1	3	3	3	2	2	1	4	4
*Chryseobacterium indologenes*	7	2.1	0	1	0	1	2	2	1	2	0	1	0	4
*C. memingosepticum*	5	1.5	0	0	0	1	2	1	1	1	2	1	1	0
*Chryseobacterium* spp.	4	1.2	0	0	0	1	1	2	0	0	0	0	0	4
*Burkholderia cepacia*	3	0.9	0	0	3	0	0	0	0	0	0	1	2	0
*Pseudomonas putida*	1	0.3	0	0	0	1	0	0	0	0	1	0	0	0
*Moraxella osloensis*	1	0.3	0	0	0	0	0	1	0	0	0	0	1	0
*Moraxella morganii*	1	0.3	0	0	0	0	0	0	1	0	0	0	0	1
**Anaerobes**	**4**	**1.2**	**0**	**1**	**0**	**0**	**0**	**2**	**0**	**2**	**0**	**0**	**0**	**2**
**Fungus**	**51**	**15.2**	**1**	**4**	**0**	**11**	**19**	**13**	**4**	**1**	**6**	**6**	**10**	**28**
**Yeast**	**45**	**13.4**	**0**	**3**	**0**	**10**	**15**	**13**	**4**	**1**	**6**	**4**	**8**	**26**
Yeast	25	7.5	**0**	1	0	8	7	9	0	0	2	2	4	17
*Candida* spp.	14	4.2	**0**	2	0	2	5	2	3	1	3	2	4	4
Others	6	1.8	0	0	0	0	3	2	1	0	1	0	0	5
**MOLD**	**6**	**1.8**	0	**1**	**0**	**1**	**4**	**0**	**0**	**0**	**0**	**2**	**2**	**2**

*MDRO* multiple drug-resistant organisms, *MRSA* methicillin-resistant *S. aureus*, *VRE* vancomycin-resistant *Enterococcus*, *CRKP* carbapenem–resistant *K. pneumonia*, *CRE*, carbapenem-resistant *Enterobacteriaceae*, *IRAB* imipenem-resistant *A. baumannii*, *PDRAB* pandrug-resistant *A. baumannii*, *PDR-PA* pandrug-resistant *P. aeruginosa*

*PDRAB was defined as being resistant to aminoglycosides, antipseudomonal carbapenems, antipseudomonal fluoroquinolones, antipseudomonal penicillins+β-lactamase inhibitors, extended-spectrum cephalosporins, folate pathway inhibitors, penicillins+β-lactamase inhibitors, polymyxins, tetracycline

*PDRPA was defined as being resistant to aminoglycosides, antipseudomonal carbapenems, antipseudomonal cephalosporins, antipseudomonal fluoroquinolones, antipseudomonal penicillins+β-lactamase inhibitors, monobactams, phosphonic acids, polymyxins

**Fig. 1:**
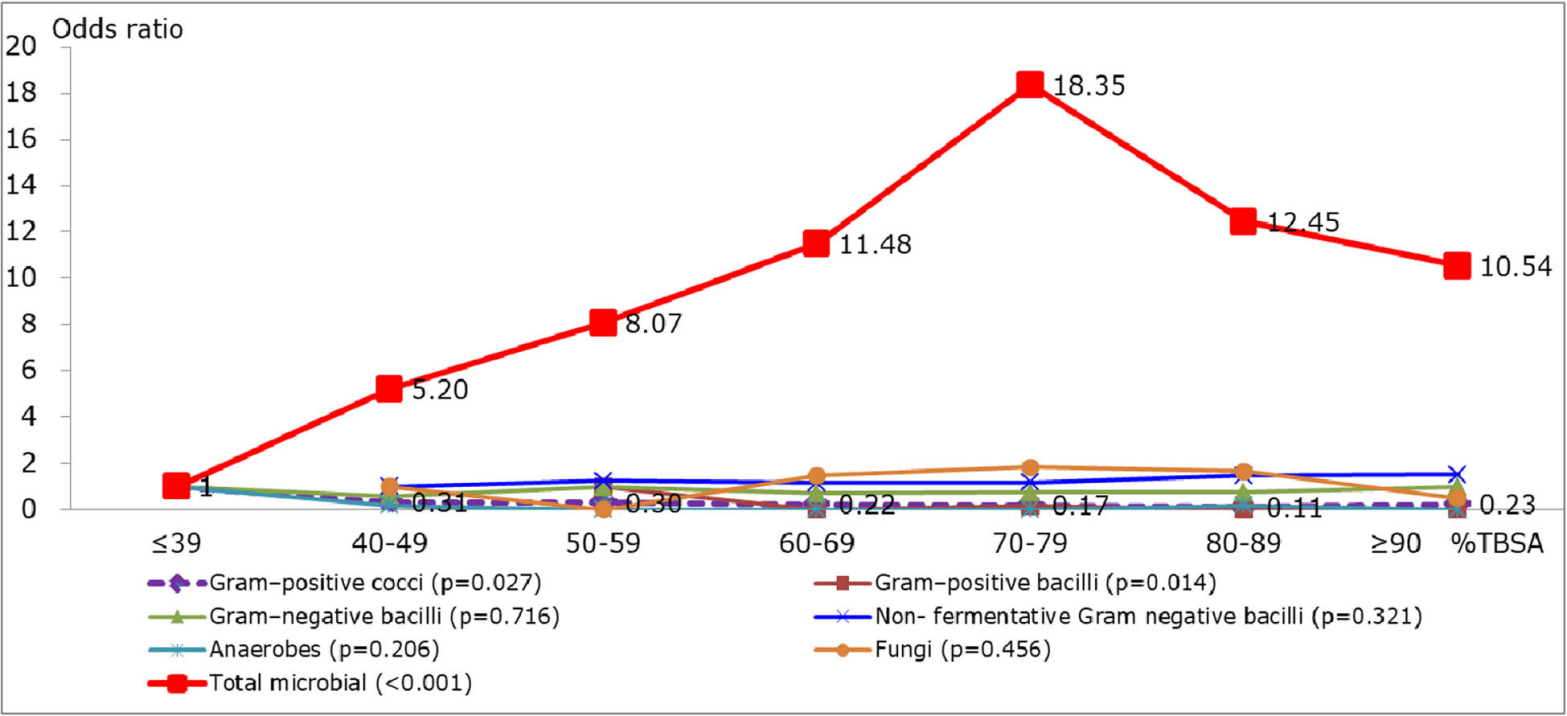
Trends in distribution of microbial isolates by total body surface area in the culture-positive specimens

Figure 1 and Table 3 shows the trends in distribution of microbial isolates by TBSA. Overall, the total number of microbial isolates gradually increased with the increase in TBSA (*p* < 0.001). The OR peaked at 70–79%TBSA (18.35), before declining to 12.45 at 80–89% TBSA. The decline in OR slowed to 10.54 at more than 90% TBSA. At 39% or less TBSA, significant change was observed for GPC (*p* = 0.027), however the OR decreased with increasing TBSA and maintained between 0.17–0.31. Gram–positive bacilli isolates (only 4 strains) first appeared at 50–59% TBSA, the OR also decreased with increasing TBSA (*p* = 0.014).

**Fig. 2:**
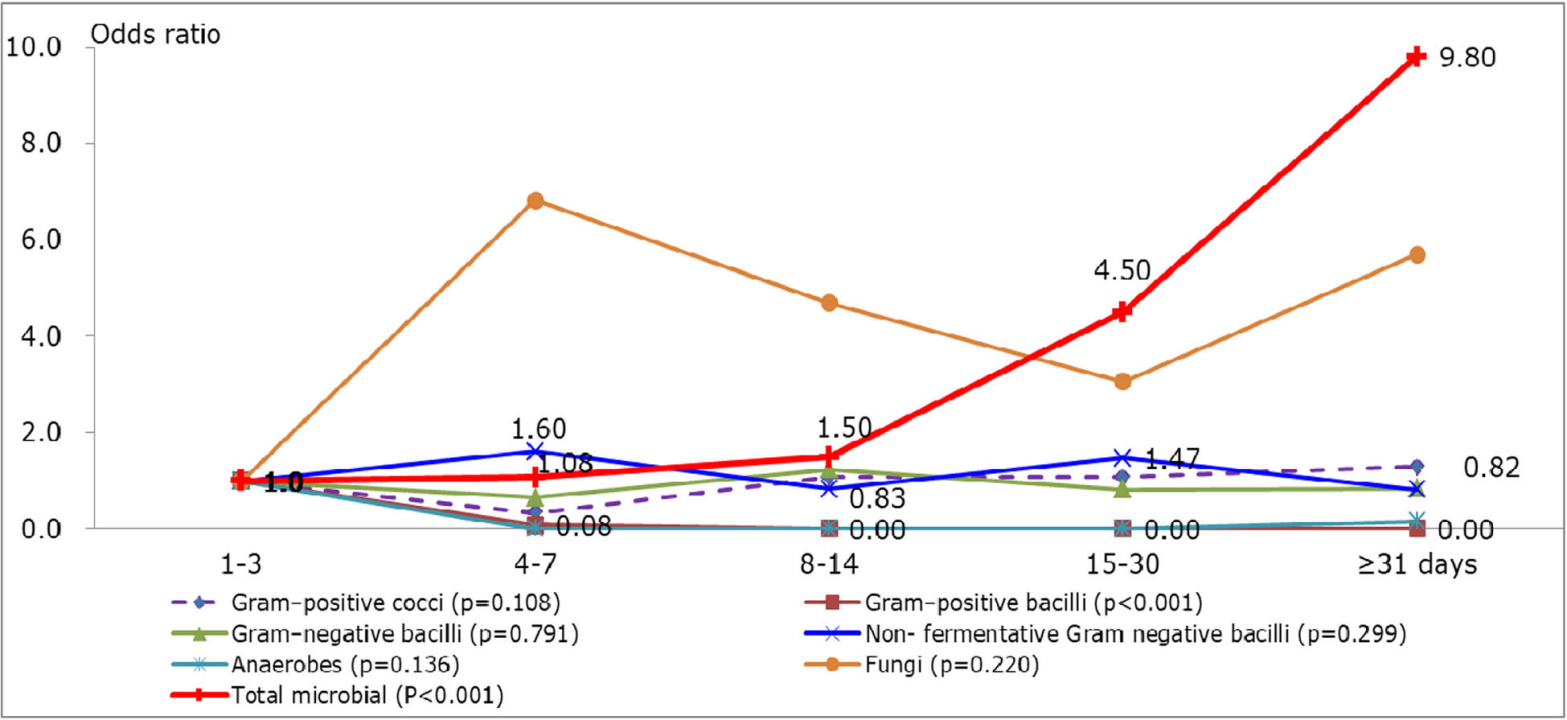
Trends in the main microbial isolates by days after admission in the hospital (Culture positive specimens)

Figure 2 indicate the trends in microbial isolation by days after admission. Overall, the total number of microbial isolates gradually increased with the increase in the hospitalization days (*p* < 0.001). The OR (4.50) of microbial isolates increased markedly in 15–30 hospitalization days and the OR (9.80) reached peak at 31 hospitalization days or more. Gram–positive bacilli showed statistically significant change (*p* < 0.001), which was the highest in 1–3 hospitalization days, but the OR of isolates decreased with increasing hospitalization days. For all other microbial isolates, such as GPC, GNB, GNFGNB, anaerobes and fungi, no statistically significant difference (*p*>0.05) in OR trends was observed. The OR (6.82) of the fungal isolates reached the highest at 4–7 hospitalization days, followed by a slight decrease but remained stable (3.05–5.69) thereafter.

### Main risk factors

Logistic and Cox regression were used to analyze potential risk factors for MDRO (Table 4). In the simple logistic and Cox models, the TBSA showed statistically significant difference (*p* = 0.003), while the third-degree burn severity showed statistically significant difference (*p* = 0.002) in the logistic model, but no statistical difference was observed in Cox Model (*p* = 0.076). In the multiple regressions, for each additional 10% of the TBSA, the MDRO increased 17.57 times (95% CI [1.47–21.0], *p* = 0.024) by logistic model and 2.58 times (95% CI [1.71–3.88], *p* < 0.001) by Cox model. For each additional 10% increase in third-degree burn severity, the MDRO decreased significantly by 47% (95% CI [0.38–0.73], *p* < 0.001) by Cox model. In addition, surgical intervention was not statistically significant in isolating MDRO (*p* = 0.057).

**Table 4:** Regression models of risk factors for multi-drug resistant microbes

Variables	Logistic regression model				Cox regression model			
	Unadjust		Adjust		Unadjust		Adjust	
	OR (95% CI)	*p*-value	OR (95% CI)	*p*-value	HR (95% CI)	*p*-value	HR (95% CI)	*p*-value
Total body surface area (every 10%)	3.6 (1.6–8.2)	.003	17.57 (1.47–21.0)	.024	1.31 (1.01–1.56)	.003	2.58 (1.71–3.88)	< .001
Third burn-degree severity (every 10%)	1.79 (1.2–2.6)	.002	0.26 (0.05–1.41)	.118	1.14 (0.99–1.32)	.076	0.53 (0.38–0.73)	< .001

*OR* odds ratio, *CI* confidence interval, *HR* hazards ratio

## Discussion

This study was conducted on young population that suffered burns due to single dust explosion event. Given high homogeneity among cases, our results demonstrated that the TBSA range and the hospitalization days were independent factors for the isolation of MDRO. However, the severity of burns and the time elapsed after burns influenced the various microbial isolates differently.

### Microbes and drug-resistant bacteria

Several reports indicated that the most common genera isolated from wound or burn units were GPC, followed by GNB [1, 2, 6, 7]. However, the results from this study differed from previously published reports. We found the most commonly isolated group was GNB (53.1%). Among GNB, the most commonly isolated subgroup was GNFGNB (39.4%). This difference could be attributed to differences in the type of burns, populations, age distribution, and data analysis period. However, the most common bacteria isolated in this study were *Acinetobacter* spp*.* and *P. aeruginosa,* which is consistent with a majority of other published reports [1, 2, 7, 9]. Interestingly, those two bacteria along with *Stenotrophomonas maltophilia* were isolated within 3 days of hospitalization. One study on similar population affected by dust explosion but where patients were transmitted to other hospitals for treatment, found that *Ralstonia pickettii* (18.5%) was one of the most common bacteria causing BSIs [20]. These bacteria survive easily in sewage or humid environments.

Among MDRO, Gram-positive bacteria particularly MRSA continues to be an important pathogen in burn infections [1, 4]. Our data showed lower frequency of isolation by *S. aureus* (11.3%) compared to MRSA (95%). MRSA cases showed TBSA of more than 40% and were hospitalized for more than 15 days. This result was in contradiction with another study that monitored all hospitalized burn patients for 1 year and found only 16.7% *S. aureus* strains resistant to methicillin. However, burn patients in that study merely had a median TBSA of 7% (interquartile range 4–14%) and a median hospital stay of 14 days [7]. Apart from MRSA, care and treatment of burn patients infected with VRE has also become increasingly challenging. Isolation rate of VRE in the present study was low (2.1%). The reason could be younger healthy population enrolled in the present study coupled with proper antibiotic management and infection control.

Gram-negative bacteria have increasingly become highly resistant to antimicrobial agents due to their ability to form biofilms that appear to enhance their pathogenicity [21]. Shoja et al. found a high rate (92.5%) of isolation of CRAB [22]. However, their report lacked important parameters such as the range and depth of burns, and the distribution of age. Our data displayed that in all types of ICU, the IRAB BSIs of burn CU increased during the first month after hospitalization. The TBSA in those cases ranged from 67 to > 90%, and those infections occurred 6–17 days after admission. More information might be provided if molecular epidemiological surveillance was available. However, the overall data from our study showed that the incidence of *A. baumannii* isolates resistant to carbapenem was relatively low, and the result was similar to the incidence of CRABs (21%) reported by two similar groups, but treated in different hospitals [23, 24]. Apart from CRAB, the resistance rates for other GNB were also quite low, such as carbapenem–resistant *Enterobacteriaceae* (0.3–2.4%) and pandrug–resistant *P. aeruginosa* (0.3%). Antimicrobial stewardship is extremely important in burn units [25]. In order to reduce microorganisms and MDRO in the environment, our study not only formulated cleaning procedures and educating cleaners, also used auditing procedures such as use of luminometers to ensure proper disinfection and cleanliness to reduce the risk of microbial and MDRO presence and transmission.

### Main risk factors for microbial colonization and infection

In the present study, besides the endogenous and exogenous microbial colonization or infection there were other risk factors that could promote microbial infection and increased resistance to antibiotics in burn patients. Due to the fact that subjects in this study belonged to a young and homogeneous group, of particular interest to us was the influence of both burn severity and time factor on the types of microbial isolates. Several multivariate analyses of prospective or retrospective studies have revealed that the degree of burn wounds was a major risk factor for microbial colonization and infection [1, 5, 7, 26]. Compared to ≤19% of TBSA, patients with greater than 20% of TBSA were more than twice (RR 2.09–2.41) likely to be infected at a rate that was five times faster [7]. Several studies revealed that patients with TBSA range greater than 25% or greater than 30% had a high risk of microbial infection, because these patients usually need to use invasive devices for treatment [4, 26]. One report suggested that infection rates rose by an increase in TBSA of burn; they observed infection rates of 18, 30 and 52% in patients with 15–30%, 31–50% and 51–69% of TBSA respectively [5]. In the present study, patients with severe burns cases had the average TBSA of 46.1%; however, infection rates for patients with greater than 50% TBSA rose by 56.8%.

Unexpectedly, our data showed the number of isolated GPC were the highest in patients with less than 39% TBSA, and the proportion of isolated GPC showed a downward trend (*p* = 0.027) with the increase in TBSA extent. However, in terms of overall isolated microorganisms, our data were consistent with previously published reports [1, 5, 7]. We found an upward trend (*p* < 0.001) in the proportion of overall microbial isolates over time, until the TBSA reached 79%. Afterwards, the trend stabilized followed by a decline. In general, the risk of death of patients with severe burns was relatively high and rapid, so overall isolated microbial maybe thereby reduced. However, our data could not confirm this statement due to death in only one case (70% TBSA). In terms of the overall MDRO, we also used regression analyses approach to adjust the depth of burns, the likelihood of infections by MDRO increased by 2.58–17.57 times for each 10% increase in TBSA.

Another important variable related to severity of burn is the burn depth. One report indicated that the third-degree burns would significantly influence (HR 7.88) the burn wound infection. They concluded that risk of infection for patients with third-degree burns almost doubled and patients got infected at a faster rate [7]. Another study using multiple analysis revealed that only the third-degree burn (OR 1.10, 95% CI, 1.02–1.19, *p* = 0.012) was an independent risk factor [23]. Using univariable analysis, our results demonstrated significantly higher likelihood of MDRO infections (1.14–1.79 times) for every 10% increase in the third-degree burn severity. However, when the multiple analyses were used after adjusted TBSA variable, the risk of MDRO was reduced by 47–74%. This may be linked to early excision, debridement and grafting, use special materials dressings to treatment, as well as appropriate antibiotic use and infection control, which could reduce the risk of MDRO and cross-contamination in patients with deep burns.

The time factor is also one of the important factors influencing colonization or infection of predominant microorganisms in burn patients [2]. Generally, early colonization of the wound in the first 48 h occur with Gram-positive bacteria from the endogenous skin flora [1]. Next, Gram-positive bacteria are replaced by Gram-negative bacteria by the second week [2]. Hereafter, if the wound closure is delayed and the patient becomes infected and is treated with broad-spectrum antibiotics, a further shift in flora towards yeasts, fungi, and antibiotic-resistant bacteria takes place [4].

Devrim et al. found that the median duration of development of BSIs caused by GPBs from the time of burn was 5 days (ranging from 2 to 54 days after burn), significantly less than that of BSIs caused by GNB (12 days) and fungal pathogens (13 days) [6]. Recently one study showed that the overwhelming majority of bloodstream pathogens were GNB isolates typically isolated within 2 weeks after the burn. During 15–28 days after burn, *A. baumannii* was the most common causative pathogen, while *Chryseobacterium* spp*.*, *S. maltophilia,* and *R. mannitolilytica* were most frequently isolated early (mean: 14.5 days) after the burn [24]. The proportion of the overall microbial isolates in this study showed a significant change in trend over time; in particular, hospitalization for more than 14 days (OR 4.5–9.8, *p* < 0.001). Our findings correlated well with previous studies with reference to isolation of GPBs; nearly a half of them (only 4 strains) were isolated within 3 days of hospitalization, before showing downward trend (*p* < 0.001). Although there was no statistically significant difference in the trend for the proportion of GNFGNB isolation (*p* = 0.299), with *Acinetobacter* spp*.* (71.4%) and *P. aeruginosa* (84.8%) being two major groups isolated at 15 or more days after hospitalization.

One of the major strengths of this study was that it adopted stratification analysis to examine trends in major microbial isolates based on the severity of the burn and time elapsed after burn. However, our study also had some limitations. First, the sample size of cases with severe burns was relatively small, although the source of the samples was single dust explosion event. The cases had homogeneity including patient age, medical treatment and care, and environmental setting factors. Therefore, our results successfully demonstrated the trends in microbial isolation and important factors that govern the rate of isolation. Second, the dispersion of the patients in different 5 ICUs and burn ward might cause some heterogeneity of the sampling. However, an interdisciplinary medical team was quickly established and held regular meetings on treatment and disposal strategies to achieve consensus and consistency. Third, this manuscript was mainly based on recommended of the US CDC’s definition of burn infection, including changes in the appearance or characteristics of burn wound. Burn injury could result in severe sepsis and septic shock; however, our database could not display the data.

Finally, biomarkers can be used to monitor various aspects of disease progression and patient health. The World Health Organization proposed a broad definition of biomarker including almost any measurement reflecting an interaction between a biological system and a potential hazard, which may be chemical, physical, or biological. Thus, the biomarkers as an indicator can be objectively measured and evaluated, such as single molecules, protein panels, injury characteristics, or clinical parameters that may affect clinical outcomes in the severely burned [27]. We suggest that future research can be explored in other aspects, including the impact of surgical intervention and antimicrobial stewardship on the type of microorganisms isolated, and perform molecular typing of microbial isolates.

## Conclusion

We found that the proportion of overall microbial isolates increased with increase in the extent of TBSA and the time elapsed after burns. The extent of TBSA was the most important factor affecting MDRO. The study demonstrated that under well-integrated and operational medical and infection control teams, microbial transmission could be reduced to improve the survival rates of burn patients.

## Data Availability

The datasets used and/or analyzed during the current study are available from the corresponding author on reasonable request.

## Abbreviations

BSI: Bloodstream infection
CI: Confidence interval
CRAB: Carbapenem-resistant *Acinetobacter baumannii*
CRKP: Carbapenem-resistant *Klebsiella pneumoniae*
ESBL: Extended-spectrum beta-lactamase
GNB: Gram-negative bacillus
GNFGNB: Glucose non-fermenting Gram-negative bacillus
GPC: Gram-positive coccus
HAI: Healthcare-associated infection
HR: Hazard ratio
ICU: Intensive care unit
MDRO: Multiple drug-resistant organism
MRSA: Methicillin-resistant *Staphylococcus aureus*
OR: Odds ratio
TBSA: Total body surface area
VRE: Vancomycin-resistant *Enterococcus*
